# Auricular prostheses attached to osseointegrated implants: multidisciplinary work-up and clinical evaluation

**DOI:** 10.1007/s00405-019-05311-0

**Published:** 2019-02-05

**Authors:** Maarten A. Vijverberg, Luc Verhamme, Pascal van de Pol, Henricus P. M. Kunst, Emmanuel A. M. Mylanus, Myrthe K. S. Hol

**Affiliations:** 1 Department of Otorhinolaryngology, Donders Center for Neurosciences, Radboud University Medical Center, PO-box 9101, Philips van Leydenlaan 15, 6500 HB Nijmegen, The Netherlands; 2 Department of Maxillo-Facial Surgery, Radboud University Medical Center, Geert Grooteplein Zuid 10, 6525 GA Nijmegen, The Netherlands; 3 Department of Dentistry, Radboud University Medical Center, Philips van Leydenlaan 25, 6525 EX Nijmegen, The Netherlands

**Keywords:** Implants, Auricle, Prosthesis, Microtia, Skin reactions, Hearing loss

## Abstract

**Purpose:**

Not long after the introduction of osseointegrated implants outside the oral cavity, auricular prostheses are retrained on osseointegrated implants. New insights have been gained with the next-generation percutaneous osseointegrated titanium implants for bone conduction hearing since its introduction in 2010. As a result, the same technology was introduced in the Vistafix^®^ system (VXI implant) to retain auricular prostheses. The aim of this study is to evaluate the surgical procedure, clinical outcome, and satisfaction of the patient of osseointegration-retained auricular prosthesis using VXI implants.

**Materials and methods:**

11 patients who received an auricular prosthesis using VXI implants between December 2012 and November 2017 were evaluated retrospectively. The patient’s medical files were reviewed to assess clinical complications and the necessity for revision surgery. The subjective outcome was measured using the Glasgow benefit inventory (GBI).

**Results:**

In total, 31 implants were placed in 11 patients. None of these implants were lost nor revision surgery needed. An adverse skin reaction was observed in 13.0% of the implants and in 27.2% of the patients, adequately treated with an antibiotic ointment. The average follow-up time was 2 years and 7 months. The GBI displayed a positive score in every patient.

**Conclusions:**

The VXI implants used are a safe and reliable treatment option for retaining auricular prostheses in patients with an absent auricle. Patients were satisfied with their auricular prosthesis and showed benefit in quality of life. Studies with larger numbers and preferably a prospective character are needed to draw statistically significant conclusions.

## Introduction

Different causes of an absent auricle exist and can mainly be divided into congenital and acquired. Congenitally, the (partially) absent auricle, i.e. microtia can arise as a result of a single, unique genetic feature or as a part of a syndrome and can be classified into several types with or without atresia of the external auditory meatus [1, 2]. Acquired forms of an absent auricle arise from traumatic events, dissatisfying reconstruction or after amputation as a result of surgical treatment of advanced cancer of the auricle and/or external auditory meatus [3, 4].

Since complete care of the auricle is advocated, hearing revalidation requires attention in patients with a congenital absent auricle. It has been demonstrated that unilateral hearing loss in children might be associated with delayed speech-language development and behavioural problems occur more frequently [5, 6]. Thereby, different treatment options should be discussed with the caretakers. Depending on the severity of the atresia, a surgical repair might be effective in terms of hearing outcomes, taking risks and complications into consideration. This seems feasible in the minority of the atresia cases. Another option is a hearing implant, i.e. a bone conduction device (BCD) or middle ear implant [7, 8]. To discuss these types of hearing implants with the caretakers requires expertise and experience. The risks and complications need to be discussed, especially in respect of the congenital anomalies. In case of revalidation of the hearing by means of a BCD, it is important to mention a percutaneous BCD is associated with the best output [9].

Whereas the hearing loss is obvious in patients with congenital atresia, hearing loss can also occur in patients with an acquired absent auricle and hearing therefore always requires attention [4, 10].

Three different approaches can be chosen regarding the management of the auricle [11]: (1) accept and wait, where the patient chooses to leave the absent auricle untreated after being informed at the auricle consultation, (2) an auricular prosthesis, the patient chooses to have his auricle reconstructed using an implant- or adhesive-retained prosthesis, (3) a reconstruction of the auricle whereby the auricle is reconstructed using autologous rib cartilage or porous polyethylene.

An auricular prosthesis is an alternative to the autologous and alloplastic reconstruction of the auricle. Advantages such as the short procedure, possible under local anaesthesia and usability in compromised tissues make this an excellent option in patients with medical comorbidities and oncological patients [12]. Also, a prosthesis is indicated after failed autologous reconstruction and can be indicated primarily in microtia, depending on the patient’s preference [13]. Nowadays, an auricular prosthesis also has a great aesthetic and realistic appearance.

To attach the auricular prosthesis, an adhesive substance or osseointegrated implants can be used. An adhesive-retained prosthesis has a few disadvantages compared to an implant-retained prosthesis: it is easily dislocated and has a chance of adverse tissue reactions [14]. The use of osseointegrated percutaneous implants has minimized these disadvantages, although the implants themselves require daily maintenance and skin infections do occur [15]. The concept of osseointegration with titanium implants was first implemented in dental medicine in 1965 by Brånemark [16]. In 1977 osseointegrated implants were introduced for the first time outside the oral cavity by Tjellström, i.e. in the temporal bone for application of bone conduction hearing and eventually for auricular prostheses in 1983 [17].

The Brånemark implants were used for many years to retain auricular prostheses. The titanium implants used for bone conduction hearing have evolved enormously since their introduction. In 2010, the most recent design percutaneous bone implant (the BIA300) was developed for bone conduction hearing. This new design with an increased bone-to-titanium surface showed advantages compared to the previous generation implants in terms of adverse skin reactions and implant stability [18, 19]. As a result, Vistafix^®^ implants (VXI implants) were developed to retain auricular prostheses based upon the same BIA300 implant. These have been used at the Radboudumc to retain auricular prostheses since 2011.

A number of studies have been published describing the clinical results of these bone-anchored hearing implants [18–21]. However, large data regarding the VXI implants are lacking. In this study, we will describe our way of working in a multidisciplinary approach in the use of osseointegration-retained auricular prostheses using VXI implants and share our experience by retrospectively evaluating the surgical procedure, clinical outcome and satisfaction of these patients.

## Materials and methods

### Patient characteristics

All patients who received an auricular prosthesis with Cochlear Vistafix VXI300 implants (Cochlear Bone Anchored Solutions AB, Mölnlycke, Sweden) at our clinic between December 2012 and November 2017 were identified. This resulted in a cohort of 11 consecutive patients with 12 auricular prostheses and a total of 31 VXI implants. The average age of the patients at implantation is 44 years and 6 months. The youngest patient was 13 years and 4 months old and the oldest patient was 85 years old at implantation. The patient characteristics are shown in Table 1. This retrospective study received approval from the local institutional ethics committee.

**Table 1:** Patient characteristics

Patient	Sex	Age	Etiology	Side	Radiotherapy	Previous surgery^a^
1	M	54	Microtia	AD	–	Yes
2	M	19	Microtia	AS	–	Yes
3	M	17	Microtia	AS	–	–
4	M	89	SCC^b^	AD	Yes	–
5	M	18	Microtia	AD	–	–
6	M	71	SCC	AS	Yes	–
7	M	52	Melanoma	AD	–	–
8	M	86	SCC	AD	Yes	–
9	M	61	Trauma	AD	–	–
10	F	18	Microtia	ADS	–	Yes
11	F	29	Microtia	AD	–	Yes

^a^Previous surgery = history of auricular reconstruction with rib cartilage in different centres

^b^SCC = Squamous cell carcinoma

### Pre-operative planning

**Fig. 1:**
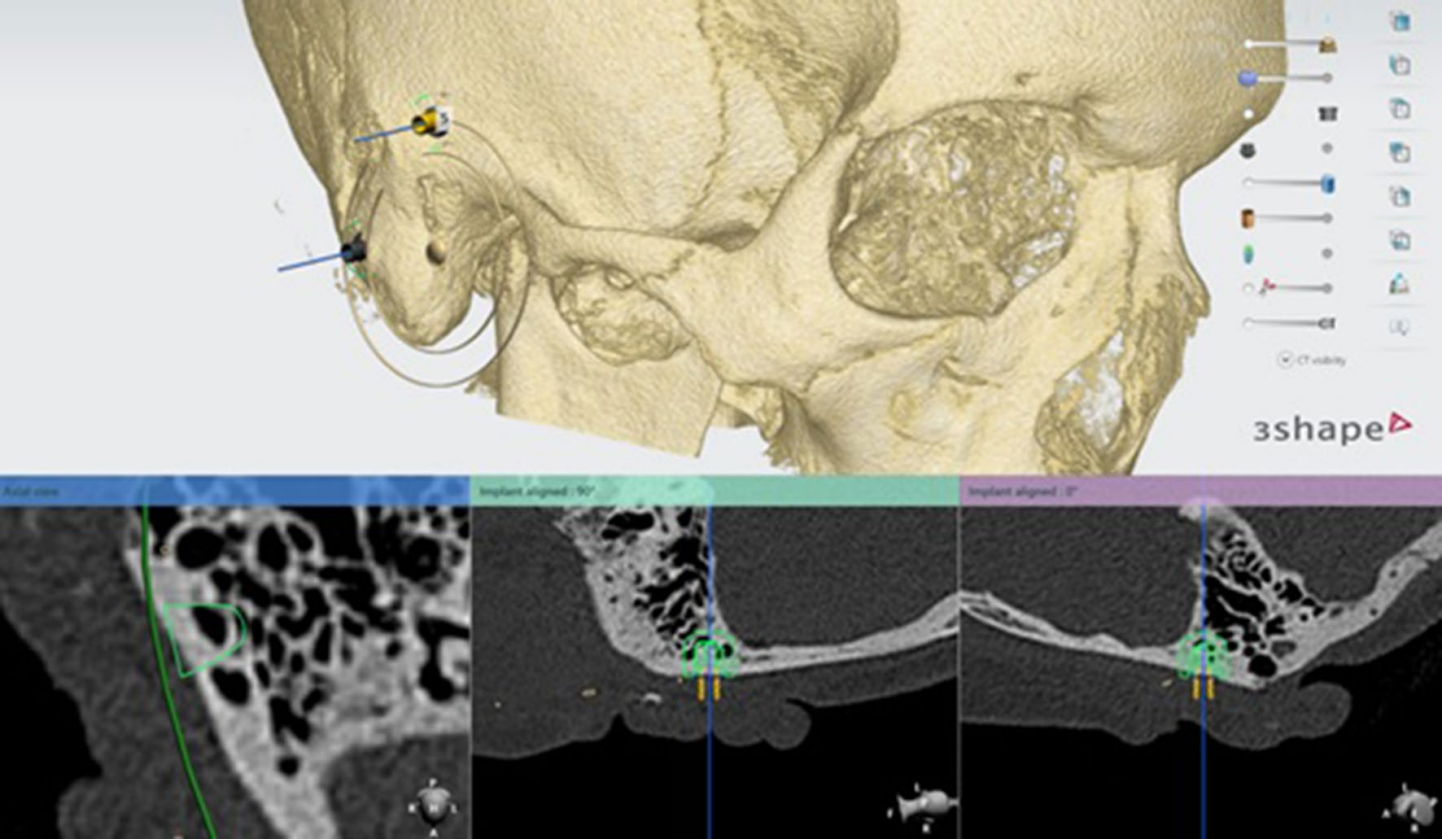
Pre-operative planning of the implants using a CT-scan

**Fig. 2:**
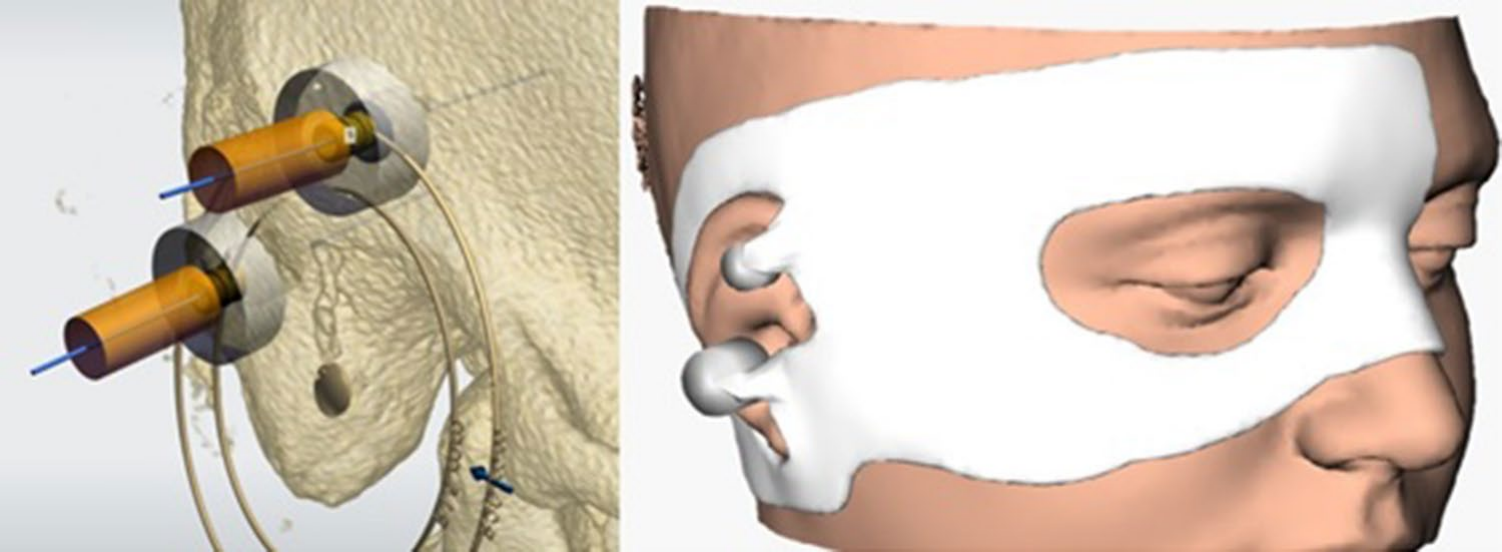
Virtual planning of implants (left) transferred to a skin template (right)

**Fig. 3:**
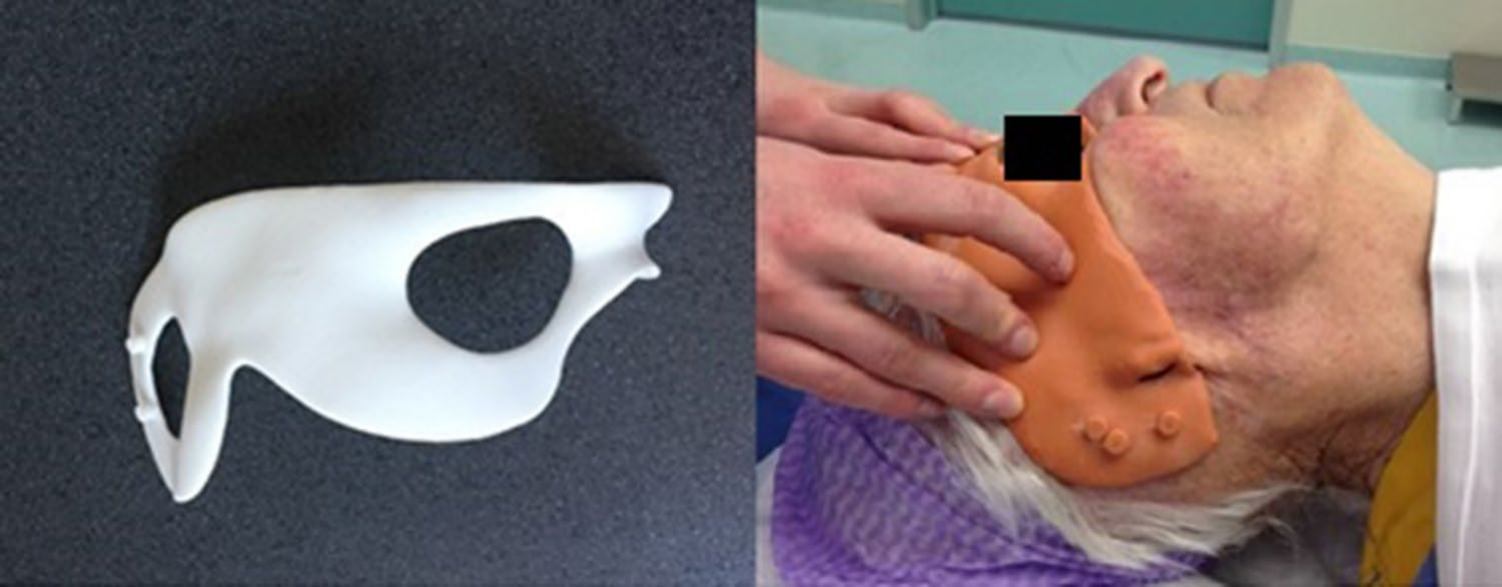
Surgical template (left) and placing over patients face pre-operatively (right)

The computed tomography (CT) scan (ossa petrosa) is used to determine the optimal location of the implants to retain the prosthesis (See Fig. 1). To define this exact location, it is important to assess if sufficient temporal bone is present at the preferred location of the implant guaranteeing a symmetric position of the prosthesis. When the ideal position of the implant is determined by the ENT-surgeon, anaplastologist and Radboudumc 3D laboratory, the virtual implant planning is transferred to a skin template which is manufactured using 3D printing (See Fig. 2). All to assure the most accurate placement of the implant during surgery (See Fig. 3). This type of template is used in all patients implanted with VXI implants in our tertiary referral center.

### Surgical technique

**Fig. 4:**
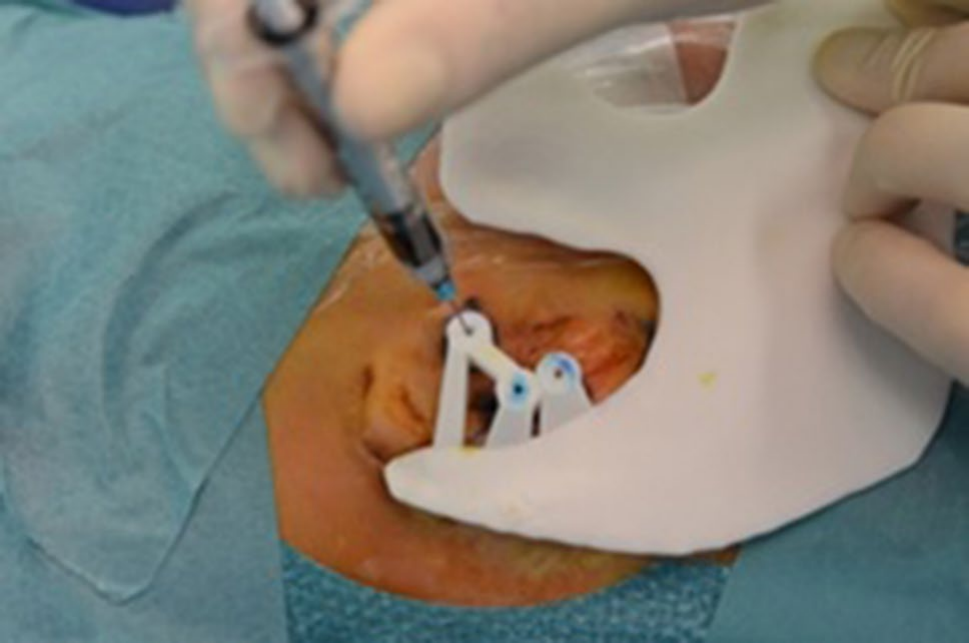
Marking of the positions of the implant using the template pre-operatively

The surgical procedure starts with shaving hair at the supposed position of the implants. The determined position of the implants is marked at the temporal bone with methylene blue using the 3D-printed skin template (Fig. 4). Methylene blue is injected with a needle through the holes. The patient’s face is completely visible. If necessary, any remnants of microtia or previous reconstruction are removed in the same session.

After a semilunar incision 10 mm posterior to the anticipated locations of the implants down to level of the periosteum, the skin and the subcutaneous flap is developed and a cruciform incision is made in the periosteal layer. Usually, three implants (at least two) are placed to guarantee optimal retention of the prosthesis.

A hole of 4 mm depth is drilled (2000 RPM, with permanent watercooling), after which a small countersink is created and the first phase of the implant is placed (torque: 45 Ncm, in case of a bony bed first a few turns without irrigation, the last turns with adequate watercooling). Before positioning of the implant, the entity of the bottom (bone or dura) is checked by palpation and visual inspection. The first implant is placed in a perpendicular direction to the bone. In patients ≤ 10 years old or when a problematic skin healing is anticipated a two-stage surgery is indicated and a cover screw is placed.

In most cases single-stage surgery can be used and Vistafix^®^ abutments (VXA abutments) are placed in the same session after all implants have been inserted. No magnets or clips were used in the studied patients. All patients received a retention bar to connect the implants. To ensure parallel positioning of the implants (and abutments), a unigrip screwdriver/implant inserter is used to extend the first implant (with the correct perpendicular position to the bone). The second and, if used, third implant are placed in parallel with the first implant and not necessarily in perpendicular position with the bone.

Following the trends in bone conduction hearing implant surgery, a shift is seen in VXI implant surgery from tissue reduction towards tissue preservation the past years [22–24]. Nowadays, in most cases the subcutis is preserved and skin is closed with resorbable sutures. Only in cases of an extensive soft-tissue layer, not enabling the use of a 7.5 mm length abutment (maximum available length), a tissue reduction is performed. Afterwards the skin surrounding the abutment is punched (5 mm biopsy punch) and the skin falls closely around the abutment. Finally, a healing abutment (6 mm) or healing cap (14 mm) and a gauze with antibiotic ointment is given followed by a compression bandage.

### Post-surgery protocol

All patients had their postoperative visit a week after surgery when the head dressings were removed. Healing caps were removed and replaced by healing abutments, which stay in position until 3–5 weeks after surgery.

Parallel to this follow-up, the patient is seen by the anaplastologist 3–5 weeks after implantation, who manufactures the auricle. At this visit, the Cochlear impression coping squared for VXA abutments are placed on the abutments and an impression is made using silicon. Next, the abutment replica’s for VXA abutments are placed on the impression copings and the impression is poured in plaster to create a replica of the situation of the patient’s head around the implants.

Using the CT scan made to manufacture the surgical template, the contralateral ear is mirrored by the 3D laboratory and printed in a plastic material (Oceanz BV, Ede, The Netherlands). A template is made of this plastic ear with silicon, which is poured with wax to make a wax ear. In case of a bilateral absent auricle, the appearance of the prosthesis is determined by the shape of the patient’s face or an impression of the ear of a relative is used.

Subsequently, this wax ear is fitted to the plaster model with the VXA abutment replica. This plaster model with abutment replica and the wax ear is sent to Pro Scan (Zonhoven, Belgium) to design and mill a titanium suprastructure, which is placed on the VXA abutment replica. A template of the suprastructure is made with silicon, which is then used to duplicate the suprastructure with epoxy die material and then poured with plaster to create a replica of the plaster template with VXA abutment replicas with the suprastructure.

The medial aspect of the wax ear is adjusted to fit Friadent Gold Bar Clips (Dentsply, Sevenum, The Netherlands) to retain de prosthesis, and the residual space is filled with a transparent plastic acryl carrier (Candulor, Rielasingen-Worblingen, Germany). The wax ear can now be attached to the suprastructure or its duplicate. Then, the titanium suprastructure is placed on the patient’s abutments and the wax ear is adjusted to fit smoothly with the patient’s soft tissue. Also, a scan of the patient’s skin is made using a spectrocolorimeter to determine the base colour of the prosthesis (e-Skin^®^; Spectromatch, Bath, UK).

The wax ear attached to the plaster model including the duplicate suprasturcture is placed in a cuvette and imbedded with plaster. This cuvette is heated and washed to remove the wax from the cuvette.

The carrier is treated with a platinum primer G611 to ensure attachment with the future silicon prosthesis.

**Fig. 5:**
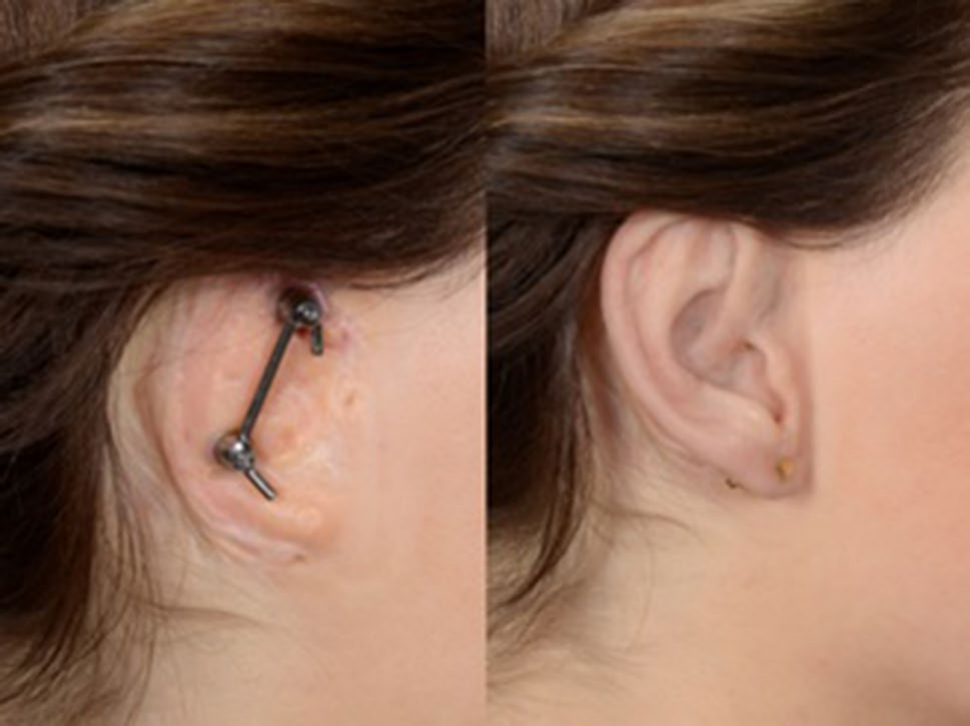
Implants (left) and prosthesis (right)

Silicon M511 A/B (Technovent, York, UK) is weighed and the colors’ ratio, determined with the spectrocolorimeter, is added. The ear is further colorized on particular locations using Short Veining Fibres P601 and flocking (Technovent, York, UK), with the patient present. This blend is placed in the plaster template in the cuvette, which is thereafter pressurized to lose the redundant silicon and air. The silicon is hardened in the oven. After some minor last adjustments, the silicon ear is ready to be placed on the patient (Fig. 5).

The implants are loaded from 3 to 5 weeks postoperatively, comparable with the early loading of the BI300 implants used for hearing rehabilitation with a bone conduction device [25]. The prosthesis is susceptible to discoloration by sunlight and therefore needs to be replaced every 1 or 2 years, depending on the patients’ needs and is taken care of within the yearly follow-up with the anaplastologist [11].

Further follow-up visits for the implant and prosthesis are scheduled after 3, 6 and 12 months followed by yearly visits in a multidisciplinary team. During these visits, implant stability was assessed manually and the surrounding skin was evaluated using the Holgers scale [26].

### Outcome measurements

To evaluate the results of the implantation with VXI implants, we studied several parameters.

Complications, i.e. implant loss, soft-tissue reactions and the necessity of revision surgery were assessed using the patients’ medical files. Also, outpatient changes in abutment length were assessed. Soft-tissue reactions were assessed using the Holgers scale [26]. A Holgers grade equal to two or higher is considered clinically relevant and requires medical treatment. Further characteristics of implantation like number of implants, depth, implant length, bottom of the drill hole, single or two-stage surgery and whether skin is reduced were assessed and are assembled in Table 2.

**Table 2:** Implant and surgery characteristics

Patient	No. of implants	Length of implants (mm)	Bottom (*N*)^a^	Length of abutments (mm)	Tissue reduction	One- or two-stage surgery
1	2	4	Bone	7.5	Yes	2
2	3	4	Bone	7.5	Yes	1
3	3	4	Dura (1) Bone (2)	6	Yes	2
4	3	4	Bone	6	Yes	1
5	3	4	Bone	7.5	No	1
6	3	4^b^	Bone	7.5	No	1
7	2	4	Bone	7.5	No	1
8	3	4^c^	Bone	7.5	Yes	1
9	2	4	Bone	7.5	Yes	1
10. AD	2	4	Bone	6	No	1
10. AS	3	4	Dura (1) Bone (2)	7.5	No	1
11	2	4	Dura (1) Bone (1)	7.5 and 6	No	1

^a^Numbers noted when not all bottoms had the same entity

^b^Inferior implant 3 mm, on which no abutment is placed

^c^No abutment was placed on anterior implant

As subjective outcome, we measured the satisfaction of the patient after implantation and loading of the prosthesis by means of the Glasgow benefit inventory (GBI) questionnaire [27]. This is an 18-item questionnaire which assesses the subjective benefit postoperatively, within four different domains: total score, general satisfaction, social benefit and physical benefit. Each question is scored using a five-point Likert scale ranging from large improvement to large deterioration. Total scores range from − 100 (maximal deterioration), 0 (no effect) to + 100 (maximal improvement).

Basic statistics were performed using IBM SPSS statistics, version 25.

## Results

### Implant and surgery characteristics

The implant and surgery characteristics are shown in Table 2.

Four patients had a history of previous auricular reconstruction surgery with autologous rib cartilage in a different center, and three patients had received radiotherapy treatment involving implant site. A total of 31 VXI implants were implanted in 11 patients, retaining 12 auricular prostheses. Depending on the availability of appropriate bone and the patients’ anatomy, the majority the prostheses were loaded on three (*n * = 7; 58%) implants, the others were based upon two (*n* = 5; 42%). All VXI implants had a length of 4 mm, except for one. This third implant was mobile during implantation. A second 3 mm implant was placed in a different position with presumed sufficient stability. It was decided not to load this specific implant with an abutment primarily, since the other two could be used. In three implants (10%) the bottom of the drillhole was dura and bony in all other implants (90%). Both 6 mm and 7.5 mm abutments were used on the implants, based on the thickness of the skin/subcutis. For most of the implants a 7.5 mm abutment was placed (*n* = 18; 58%). A 6 mm abutment was placed on 11 implants (35%) and on two implants (7%) no abutment was placed. The first case was described above (3 mm implant), and in the second case it was decided to place a third implant fixture as a sleeper to anticipate on possible future implant loss as a result of the postoperative radiation therapy.

In the surgical procedures for seven prostheses, the tissue around the implants was preserved (58%), while soft-tissue reduction was performed around the implants in five prostheses (42%). In all cases the VXI implants were implanted using single-stage surgery, except for two cases (17%). In one case a single-stage surgery was not possible due to absence of an instrument to attach the abutment to the implant. In the other case a two-stage surgery was chosen above a single-stage surgery in consultation with the patient. The patient did not want to lose his skin adhesive prosthesis during the time the implants could not be loaded.

### Post-surgery outcome

During the follow-up, no implants were lost, no revision surgery was required and there was no change in abutment length. Minimal follow-up time was 7 months and the average follow-up time was 2 years and 7 months. Three of the patients who had their auricle amputated due to a malignancy had received radiotherapy treatment prior to the implantation of the VXI implants. One of these three patients received additional radiotherapy treatment post implantation.

The patients visited the outpatient clinic for postoperative follow-up 30 times in total where an assessment of the implant and soft tissue is done, resulting in 91 observations in total. Soft-tissue reactions were recorded using the Holgers classification. A Holgers grade 0 was observed in 70.3% of the visits and a Holgers grade 1 in 8.8%. A Holgers grade 2 was observed in 16.5% and a Holgers grade 3 in 4.4%. No Holgers grade 4 was observed.

A Holgers grade 2 soft-tissue reaction or higher is noted as an adverse soft-tissue reaction and an indication for treatment with at least a locally applied antibiotic and corticosteroid ointment. This was observed at least once in ten implants (32.2%), during 19 observations (20.9%) and in at least three patients (27.2%), two of which had undergone tissue reduction, one patient with tissue preservation during implantation. Within the three patients who received pre-amputation radiotherapy, in one patient a Holgers grade 2 was observed once.

All skin reactions resolved after topical treatment with antibiotic/corticosteroid ointment. In two patients, the Holgers score was determined by how the skin around the implant was clinically looking while reviewing the files.

In the total follow-up time of 29.6 years, 24 prostheses were manufactured. The average lifespan of one prosthesis thus amounts to 1.2 years in the studied patients. The post-surgery outcome is shown in Table 3. The distribution of the maximum Holgers score observed per prosthesis is shown in Table 4.

**Table 3:** Post-surgery outcome

Patient	Implant loss	Max Holgers Score	Revision surgery	Length of abutment changed	Follow-up length (in years)	No. prostheses
1	–	0	–	–	5.13	3
2	–	2	–	–	4.21	3
3	–	1	–	–	4.06	2
4	–	2	–	–	3.96	1
5	–	3	–	–	3.81	4
6	–	0	–	–	2.13	2
7	–	0	–	–	1.58	1
8	–	1	–	–	1.55	2
9	–	0	–	–	0.85	1
10. AD	–	0	–	–	0.84	2
10. AS	–	0	–	–	0.84	2
11	–	0	–	–	0.59	1

**Table 4:** Distribution maximum Holgers Score

Maximum Holgers Score	Population (*n* = 12)^a^
Grade 0	7 (58.3%)
Grade 1	2 (16.7%)
Grade 2	2 (16.7%)
Grade 3	1 (8.3%)
Grade 4	0

^a^Total of 10 unilateral and 1 bilateral prostheses

### Subjective outcome measurements

All 11 patients were asked to fill out the GBI, which was sent by e-mail or was filled in during follow-up visits. The patients filled out the GBI at various follow-up lengths. Ten patients responded (response rate 91%). Every patient that filled in the questionnaire reported a positive score. The GBI displayed a positive change in overall health status with an average score of 25.8 (median 30.6; range 8.3–36.1). The average scores in the general domain were 38.4 (median 45.8; range 16.7–55.6), in the social domain 0 and in the physical domain − 3.3 (median 0; range − 16.7 to 0).

## Discussion

### Main findings

In this study, we have evaluated the surgical procedure, clinical outcome and satisfaction of the patient of osseointegration-retained auricular prostheses using VXI implants. Clinical outcome was assessed by complications and subjective outcome was measured using the GBI.

During the follow-up with an average of 2 years and 7 months no implants were lost, implying an implant survival rate of 100%. Three patients (ten implants) received local treatment because of an adverse soft-tissue reaction (Holgers 2 or higher). Two of these patients received treatment once during the follow-up, while the other patient received treatment four times. Revision surgery or a replacement of abutments was not needed during the follow-up.

All patients who filled in the questionnaire reported a benefit in quality of life after implantation and receiving their prosthesis (average score 25.8). It should be noted that none of the patients reported no benefit nor disadvantage in the social domain (all scores on specific questions were 0) and that two patients (18%) reported a disadvantage (− 3.3) in the physical domain after implantation (the rest reported 0 on the specific questions). This suggests that wearing the prosthesis has no adverse effects or benefit in the social domain of the patients, and there might be a slight physical disadvantage wearing the prosthesis.

### Comparison with other studies

#### Clinical outcome

In other studies conducted in patients with implant-retained auricular prostheses, an adverse soft-tissue reaction, requiring treatment, was reported in 3.5–10.4% of the observations [28–31]. Jacobsson et al. reports adverse tissue reactions in 9.68% of the observations in a paediatric population [31]. Tzortzis et al. reports a higher incidence of adverse soft-tissue reactions in children compared to adults (Holgers grade 2 or higher, 28 vs. 3% of the patients) [32].

The frequency of an adverse tissue reaction in our studied group (27.2% of the patients, 20.9% of the observations) is possibly higher due to the proportion of paediatric patients (36.3% of the patients in this study). The limited number of patients could be another explanation for the differences in frequency.

Until recently, all soft-tissue reactions were observed using the Holgers scale in our clinic. Since a new scale was designed to assess the soft-tissue reaction more objectively, the IPS-scale [33], we recommend to use this IPS scale in VXI implants as well. This scale comprises of three different parts: inflammation, pain, and skin height/ skin numbness. Depending on the different scores, a standardised treatment advice is proposed. This results in a complete and objective assessment of reporting soft-tissue reactions after implantation.

A remarkable outcome is the fact that revision surgery was not needed in our patients whereas in the literature, also in bone-anchored hearing, the reported necessity for revision surgery varies considerably, even up to approximately one-third of the patients [28, 31, 32, 34]. This might be associated with different surgical techniques (tissue preservation vs. tissue reduction [22–24]). The number of patients described with both techniques in this study is, however, too small to draw firm conclusions.

A tendency is seen towards the favourable outcomes after tissue-preservation techniques. The tissue-preservation technique used in the majority of the studied patients, may lead to less revision surgery, similar to the effects seen in temporal bone implants for bone-anchored hearing [22–24]. During the implant surgery, the skin is either preserved or reduced (6 patients vs. 5 patients, respectively). The surgical technique for osseointegrated bone-anchored hearing implants used to consist of one step where subcutaneous tissue is removed to the level of the periosteum. Since wider-diameter implants have higher survival rates enabling higher abutment lengths, tendency is to preserve this subcutaneous tissue. Studies show a better clinical outcome compared to the removal of subcutaneous tissue and is easier and faster [22–24, 35].

Future prospective and large-scale studies are needed to strengthen and supplement the clinical outcomes in the current retrospective evaluation with osseointegrated implants used to retain auricular prostheses.

#### Subjective outcome

The results on the GBI are comparable to other studies which used the GBI to assess benefit after prosthesis or alloplastic auricular reconstruction [28, 36, 37]. Kievit et al. reported an average score of 22.5 in patients with auricular prosthesis and an average score of − 1.7 in the physical domain [28]. Braun et al. reported an average score of 21.2 in patients with auricular reconstruction using a porous polyethylene implant [37]. However, a higher score is reported in autologous auricular reconstruction. Soukup et al. reported an average score of 48.1 in patients with auricular reconstruction using costal cartilage [36]. We do not have an explanation for this remarkable difference. Our hypothesis was that the average scores would be similar between the different techniques. The score reported by Soukup et al. is similar to the score reported score after rhinoplasty in adolescents, where the average score was 53.8 [38].

### Limitations

This study has a few limitations, which are important to address. First, retrospective studies are known to be prone to missing information while reviewing patient’s files. In this studied group, the Holgers score was not noted in two cases in which the maximum Holgers score was determined by how the skin around the implant was clinically looking. Second, due to the low incidence of an absent auricle among Dutch patients and the introduction of VXI implants after 2010, the sample size of this studied group is small in an absolute manner. Relatively, however, most patients in need of a prosthesis are treated in our center. To assess clinical and subjective outcome more accurately and to be able to draw firm conclusions, bigger sample sizes are preferable.

### Pre-operative 3D planning

In all patients, the placement of the implant was preceded by a CT scan to determine the optimal location of the implants and a template was manufactured to assure the most accurate placement. With the use of this pre-operative planning and surgical template a higher accuracy and precision can be achieved in positioning of the implants [39]. This facilitates the anaplastologist to design the auricle prosthesis in an optimal way, without having to compromise on the position of the fixation clips at the medial aspect of the prosthesis. Thus, the auricle will have an appealing shape and facial position, with optimal protrusion and ventilation.

### Implant loading

After the implantation of the studied VXI implants, the implants were loaded after 3–5 weeks. Originally, the time between implantation and implant loading was about 3–6 months to ensure completion of the osseointegration progress and implant stability. Recently, studies have been published about early implant loading in BIA300 implants for hearing revalidation. These studies show that loading the implant after 3–4 weeks postoperatively is safe and does not result in more implant loss or osseointegration failure [25, 40, 41]. These data in patients with implants retaining bone conduction devices resulted in the tendency to shift to early loading of the implants for retaining prosthesis. The fact no implants were lost in the patients in this study is encouraging, however, further clinical studies are needed to demonstrate if the results are consistent in osseointegrated implants used to retain auricular prostheses.

### Auricular prostheses in cancer patients

An auricular prosthesis can also be a part of the treatment in patients with ablative surgery as a result of any form of cancer or trauma. However, where some patients are happy the cancer is treated and accept the absence of the auricle, other patients would like to have their auricle reconstructed. Reconstruction with an auricular prosthesis is recommended in these patients [12]. The treatment of patients with a carcinoma of the auricle or external auditory meatus usually consists of accessory radiotherapy in the destined region of implantation. Is has previously been described that radiotherapy implies higher risks of complications and implant loss when implantation follows radiotherapy in the craniofacial area [42]. Therefore, some clinicians advise to place implants (without abutment) at the same time as ablative surgery, to obtain sufficient osseointegration [43]. We advocate a patient-orientated perspective, where our first goal is to create a safe situation without carcinoma in the temporal bone. The possibility of ear reconstruction with an auricular prosthesis is discussed after the patient is recovered, both psychologically as physically. This approach improves quality of care advocating shared decision-making and optimal implant positioning can be guaranteed. Our experience with the currently used wider-diameter implants, mainly used in bone-anchored hearing implants, as well as the encouraging results of 3 out of 11 patients in this population, is that they are safe to use after radiotherapy, which is also suggested in the literature [44].

## Conclusion

This study describes the work-up of patients with a missing auricle opting for implant-retained prostheses. No implants were lost in our total cohort of patients with an absent auricle due to microtia, trauma or cancer of the auricle and/or external auditory meatus. No revision surgery was needed in an average follow-up period of more than 2.5 years. The VXI implants used are a safe and reliable treatment option for retaining auricular prostheses in patients with an absent auricle. Adverse skin reactions appeared in 32.2% of the implants and in 27.2% of the patients, resolving after treatment with an antibiotic ointment. In general, patients were satisfied with their auricular prosthesis and showed benefit in quality of life. Further studies with larger numbers and preferably a prospective character is needed to draw statistically significant conclusions.

## Abbreviations

VXI implants: Vistafix^®^ implants
VXA abutments: Vistafix^®^ abutments
CT: Computed tomography
BCD: Bone conduction device
GBI: Glasgow benefit inventory
